# Comparative in vitro activity of piperacillin-tazobactam and temocillin against third-generation cephalosporin-resistant, carbapenem-susceptible Escherichia coli and Klebsiella pneumoniae

**DOI:** 10.3205/id000077

**Published:** 2021-12-21

**Authors:** Michael Kresken, Yvonne Pfeifer, Guido Werner

**Affiliations:** 1 Antiinfectives Intelligence GmbH, Cologne, Germany; 2 Rheinische Fachhochschule Köln gGmbH, Cologne, Germany; 3 Robert Koch Institute, FG13 Nosocomial Pathogens and Antibiotic Resistance, Wernigerode, Germany

**Keywords:** Enterobacteriaceae, ESBL, CTX-M, Germany

## Abstract

Carbapenems are considered the drugs of choice for first-line treatment of severe infections caused by carbapenem-susceptible, extended-spectrum β-lactamases (ESBL)-producing Enterobacterales, while piperacillin-tazobactam has been recommended as an alternative for treatment of non-severe infections. Temocillin is stable to ESBL and AmpC enzymes and may thus represent another treatment option. This study assessed the in vitro activity of piperacillin-tazobactam and temocillin against third-generation cephalosporin (3GC)-resistant *Escherichia coli* and *Klebsiella pneumoniae*, as compared to 3GC-susceptible isolates of either species.

One hundred and nine isolates from hospitalized patients with bloodstream and urinary tract infections were tested. All isolates were collected during the resistance surveillance study of the Paul-Ehrlich-Society for Chemotherapy in 2016/17. Minimum inhibitory concentrations (MICs) were determined by broth microdilution according to the standard ISO 20776-1 and interpreted using EUCAST clinical breakpoints (version 11.0).

Seventy-nine isolates (*E. coli*, n=58; *K. pneumoniae*, n=21) were 3GC-resistant and 30 (*E. coli*, n=15; *K. pneumoniae*, n=15) were 3GC-susceptible. Susceptibility to piperacillin-tazobactam was detected in 93.3% of 3GC-susceptible isolates (for both *E. coli* and *K. pneumoniae*) and in 79.3% and 57.1% of the 3GC-resistant *E. coli* and *K. pneumoniae*, respectively. In contrast, 3GC-susceptible isolates were 100% susceptible to temocillin as were 94.8% and 90.5% of the 3GC-resistant *E. coli* and *K. pneumoniae*, respectively.

In conclusion, temocillin demonstrated potent in vitro activity against carbapenem-susceptible, 3GC-resistant *E. coli* and *K. pneumoniae* from bloodstream and urinary tract infection samples, with susceptibility rates exceeding those of piperacillin-tazobactam.

## Introduction

Resistance to third-generation cephalosporins (in Germany also referred to as group 3 cephalosporins in accordance with the nomenclature of the Paul-Ehrlich-Society for Chemotherapy; 3GC) in Enterobacterales isolates has emerged over the past 20 years in Germany, Europe and worldwide. 3GC resistance in *Escherichia coli* and *Klebsiella pneumoniae* is most commonly mediated by extended-spectrum β-lactamases (ESBLs), but also occurs through other mechanisms like plasmid-encoded AmpC-type enzymes [1], [2]. Moreover, 3GC-resistant isolates of either species are normally also frequently resistant to non-β-lactam antibiotics such as fluoroquinolones, aminoglycosides or trimethoprim-sulfamethoxazole [3]. Group 1 carbapenems (meropenem, imipenem) are considered the drugs of choice for first-line treatment of infections caused by ESBL-producing Enterobacterales [1], [2], [3], [4]. However, their use should be limited to the treatment of severe infections in order to halt the worrying worldwide increase in carbapenem consumption [5], which already led to high rates of carbapenem resistance in *K. pneumoniae* isolates in some geographic regions [6].

Piperacillin-tazobactam at appropriate exposure (4.5 g every 6 h or 4.5 g every 8 h in extended infusion) has been recommended as an alternative to group 1 carbapenems for treatment of non-severe infections due to ESBL-producing Enterobacterales [3]. Temocillin is stable to most types of β-lactamases, including ESBLs and AmpC-type enzymes, and thus represents another possible option for the treatment of 3GC-resistant Enterobacterales infections [3]. The drug, developed in the 1980s and currently available in four member states of the European Union (Belgium, France, Germany, Luxembourg) as well as in Iran and the United Kingdom, is indicated for parenteral therapy of bloodstream infections, complicated urinary tract infections, lower respiratory tract infections and wound infections [7].

The present study assessed the in vitro activity of temocillin and piperacillin-tazobactam against a panel of selected carbapenem-susceptible, 3GC-resistant *E. coli* and *K. pneumoniae*, as compared to 3GC-susceptible isolates of either species.

## Methods

### Sampling sites and bacterial isolates

Bacterial strains tested were non-duplicate clinical isolates obtained from hospitalized patients with bloodstream or urinary tract infections, and collected at 22 medical laboratories in Germany (n=20), Switzerland (n=1) and Austria (n=1) participating in the resistance surveillance study performed by the Working Party Antimicrobial Resistance of the Paul-Ehrlich-Society for Chemotherapy from October 2016 to March 2017. Inclusion of one isolate per species and patient was permitted.

### Species identification, susceptibility testing and β-lactamase detection

Verification of species identification and susceptibility testing were performed at a central laboratory (Antiinfectives Intelligence GmbH). Species identification was verified by matrix-assisted laser desorption ionization-time of flight (MALDI-TOF) mass spectrometry (MALDI Biotyper, Microflex, Bruker Daltonik GmbH, Bremen, Germany).

Minimal inhibitory concentrations (MICs) were determined using the broth microdilution method (BMD) with geometric twofold serial dilutions of the antimicrobial agents in cation-adjusted Mueller-Hinton broth according to the standard ISO 20776-1 [8]. The final inoculum ranged between 2x10^5^ CFU/mL and 8x10^5^ CFU/mL. Antibacterial agents tested were as follows (concentration ranges in parentheses): ampicillin (0.5–32 mg/L), amoxicillin-clavulanic acid (0.5–128 mg/L), piperacillin-tazobactam (1/4–64/4 mg/L), temocillin (0.25–256 mg/L), cefuroxime (0.125–16 mg/L), cefotaxime (0.125–16 mg/L), cefotaxime-clavulanic acid (0.125/4–16/4 mg/L), ceftriaxone (0.125–32 mg/L), ceftazidime (0.25–32 mg/L), ceftazidime-clavulanic acid (0.25/4–32/4 mg/L), cefepime (0.25–64 mg/L), imipenem (0.5–64 mg/L), meropenem (0.063–32 mg/L), amikacin (0.5–64 mg/L), gentamicin (0.25–32 mg/L), tobramycin (1–16 mg/L), ciprofloxacin (0.063–16mg/L), levofloxacin (0.063–16 mg/L), colistin (1–16 mg/L), fosfomycin (1–2048 mg/L), cotrimoxazole (trimethoprim:sulfamethoxazole in the ratio 1:19, 0.25/4.75–16/304 mg/L). Industrially manufactured trays containing the antibiotics (MICRONAUT-S PEG) with one exception (temocillin), were purchased from Merlin Diagnostika GmbH, Bornheim, Germany. BMD panels of temocillin were prepared in-house. Reference strain *E. coli* ATCC 25922 was used for quality control. The MICs were read visually and interpreted according to the clinical breakpoints approved by the European Committee on Antimicrobial Susceptibility Testing (EUCAST, version 11.0, January 2021): S (susceptible, standard dosing regimen), I (susceptible, increased exposure), and R (resistant) [9].

The phenotypic and genotypic test methods used to detect ESBLs and AmpC-type enzymes have already been described elsewhere [10].

### Data processing and statistical evaluation

Data were processed using Microsoft Excel. Statistical significance of differences in resistance rates was judged by comparing 95% confidence intervals (95% CI). Intervals were constructed using the Newcombe-Wilson method without continuity correction. If neither rate was contained in the CI of the other one, significance of p<0.05 was assumed. No further statistical analysis was performed, considering the descriptive nature of the study.

## Results

### Characteristics of isolates

This study included 109 clinical isolates (*E. coli*, n=73; *K. pneumoniae*, n=36). Forty-seven and 62 isolates were sampled from blood and urine, respectively. Twenty-two of the 109 isolates (20.2%) were recovered from patients in intensive care units. 

Meropenem inhibited all isolates at the lowest concentration tested (i.e., 0.063 mg/L). Seventy-nine isolates were 3GC-resistant (*E. coli*, n=58; *K. pneumoniae*, n=21) and 30 were 3GC-susceptible (*E. coli*, n=15; *K. pneumoniae*, n=15). Of the 3GC-resistant isolates, 61 were resistant to both cefotaxime and ceftazidime, 16 were cefotaxime-resistant only (ceftazidime-susceptible [S+I]) and two were ceftazidime-resistant only (cefotaxime-susceptible [S+I]).

ESBL production was confirmed in 55/58 *E. coli* and in 20/21 3GC-resistant *K. pneumoniae* isolates, of which 74 produced different CTX-M enzymes and one *K. pneumoniae* isolate harboured the ESBL variant SHV-12. The remaining four isolates included an *E. coli* producing the AmpC enzyme DHA and an *E. coli* with a mutation in the promoter of the naturally occurring *ampC* gene, leading to increased gene expression. Overexpression of SHV-1 was presumed in one isolate each of *E. coli* and *K. pneumoniae* [10].

### Susceptibility of isolates to piperacillin-tazobactam and temocillin in comparison to the other antimicrobials tested

MIC distribution data of temocillin and piperacillin-tazobactam are presented in Table 1 (Tab. 1). For both species and for each antibiotic tested, the MICs inhibiting 50% and 90% of the isolates (MIC_50_, MIC_90_) and the number and percentage of isolates classified as susceptible (S or I) or resistant were calculated.

Susceptibility to piperacillin-tazobactam (MIC≤8 mg/L) and temocillin (MIC≤16 mg/L) among the 30 3GC-susceptible isolates was 93.3% and 100%, respectively, for both *E. coli* and *K. pneumoniae*. MIC_50/90_ values of piperacillin-tazobactam and temocillin were ≤1/4 mg/L and 4/16 mg/L, respectively, for *E. coli*, and ≤1/8 mg/L and 2/8 mg/L, respectively, for *K. pneumoniae*. One isolate each of *E. coli* and *K. pneumoniae* had a piperacillin-tazobactam MIC of ≥128 mg/L. MICs of ceftazidime for both isolates were 1 mg/L as compared to MICs of ≤0.25–0.5 mg/L for the other 28 3GC-susceptible isolates.

Susceptibility data of the 3GC-susceptible *E. coli* and *K. pneumoniae* to the other antibiotics tested are displayed in Attachment 1, Supplementary Table 1 and Attachment 1, Supplementary Table 2, respectively. Highest levels of drug resistance in 3GC-susceptible *E. coli* were observed for ampicillin (53.3%), cotrimoxazole (26.7%), and amoxicillin-clavulanic acid (20%), while the highest level of drug resistance in 3GC-susceptible *K. pneumoniae*, except for ampicillin, was observed for fosfomycin (33.3%). Two 3GC-susceptible *K. pneumoniae* were colistin-resistant.

Susceptibility to piperacillin-tazobactam and temocillin among 3GC-resistant *E. coli* was 79.3% (95% CI: 68.9–89.7%) versus 94.8% (95% CI: 89.1–100%), respectively, and among 3GC-resistant *K. pneumoniae* 57.1% (95% CI: 44.4–69.8%) versus 90.5% (95% CI: 83.0–98.0%), respectively. MIC50/90 values of piperacillin-tazobactam and temocillin were 2/32 and 8/16 mg/L mg/L, respectively, for *E. coli*, and 8/≥128 mg/L and 8/16 mg/L, respectively, for *K. pneumoniae*. Seventeen 3GC-resistant isolates (*E. coli*, n=10; *K. pneumoniae*, n=7) were resistant to piperacillin-tazobactam, but susceptible to temocillin, while one *E. coli* isolate was temocillin-resistant, but susceptible to piperacillin-tazobactam. Two isolates of either species were resistant to both antimicrobials (Table 2 (Tab. 2)).

Susceptibility data of the 3GC-resistant, carbapenem-susceptible *E. coli* and *K. pneumoniae* to the other antibiotics tested are displayed in Attachment 1, Supplementary Table 3 and Attachment 1, Supplementary Table 4, respectively. Extremely high levels of resistance (>70%) were observed for ciprofloxacin, levofloxacin and cotrimoxazole in 3GC-resistant *E. coli* and for ciprofloxacin and cotrimoxazole in 3GC-resistant *K. pneumoniae*. Furthermore, very high levels of resistance (>50%) were detected for levofloxacin, tobramycin and fosfomycin in 3GC-resistant *K. pneumoniae*. One 3GC-resistant isolate each of *E. coli* and *K. pneumoniae* was colistin-resistant, and two 3GC-resistant *E. coli* were fosfomycin-resistant.

## Discussion

Resistance to 3GC in *E. coli* and *K. pneumoniae* has become widespread in all parts of the world. According to data of the European Antimicrobial Resistance Network (EARS-Net), investigating invasive isolates, resistance to 3GC in *E. coli* rose from 1.7% in 2002 to 15.1% in 2019. 3GC resistance also increased in *K. pneumoniae*. EARS-Net reported an overall resistance rate of 31.3% for 2019, with extremely large differences in the level of resistance between individual countries (to >60%) [11], [12]. The study of the Working Party “Antimicrobial Resistance” of the Paul-Ehrlich-Society for Chemotherapy found that in 2016/17, resistance rates to cefotaxime in *E. coli* and *K. pneumoniae* were 20.1% and 16.2%, respectively. The frequency of cefotaxime resistance has risen sharply when compared to the results of previous studies performed by the Working Party. In 2001 and 2010, cefotaxime resistance rates were 2.6% and 17.4%, respectively, for *E. coli*, and 9.7% and 16.9%, respectively, for *K. pneumoniae* [13]. Temocillin demonstrated potent in vitro activity against carbapenem-susceptible, 3GC-resistant isolates of *E. coli* and *K. pneumoniae*. Data on the in vitro activity of temocillin against these isolates with a differentiation according to the ESBL types have been published elsewhere [10]. The MIC50/90 values of temocillin found in the present study (8/16 mg/L for either species) were comparable to those found by other investigators [14], [15], [16]. Susceptibility rates of temocillin exceeded those of piperacillin-tazobactam for either species. Applying the current EUCAST criteria for interpretation of temocillin MICs, 94.8% of the 3GC-resistant *E. coli* and 90.5% of the 3GC-resistant *K. pneumoniae* in the present study were susceptible, while susceptibility rates of piperacillin-tazobactam were approximately 15% (*E. coli*) and 30% (*K. pneumoniae*) lower. Temocillin may thus represent a valuable alternative to piperacillin-tazobactam for target treatment of infections caused by carbapenem-susceptible, 3GC-resistant *E. coli* and *K. pneumoniae*. Three 3GC-resistant isolates of *E. coli* and two 3GC-resistant isolates of *K. pneumoniae* were found to be temocillin-resistant, with MICs of 32–64 mg/L being 1–2 steps above the EUCAST clinical breakpoint of 16 mg/L. Several β-lactamase genes were detected in each of these five isolates (Table 2 (Tab. 2)), but the mechanisms of resistance to temocillin were not examined in this study. However, it was noticeable that temocillin MICs of 3GC-resistant isolates tended to be higher than those of 3GC-susceptible isolates (Table 1 (Tab. 1)). Seventy-five out of the 79 3GC-resistant isolates produced an ESBL, including the five temocillin-resistant strains (Table 2 (Tab. 2)). Thus, one could speculate that the combination of ESBLs and other β-lactamases might be partly responsible for “low-level” resistance to temocillin. However, a recent French study, comparing four pairs of isogenic clinical isolates of *E. coli* (one each temocillin-susceptible [MICs 2–8 mg/L] and one temocillin-resistant [MICs 32–64 mg/L]) without resistance to other β-lactams, found mutations in several genes, including a porin-encoding gene (ompD-like) and genes encoding for transport/membrane proteins [17]. The EUCAST breakpoint of temocillin, however, applies only to urinary tract infections due to *E. coli*, *Klebsiella *spp. (except *Klebsiella aerogenes*) and *Proteus mirabilis*, but also includes complicated and more severe urinary tract infections as well as urosepsis, but not severe sepsis and septic shock [18]. It must also be stressed that susceptible isolates of the relevant species fall in the I-group (I = susceptible, increased exposure), which means that the high-exposure dosing regimen of 2 g every 8 h is needed for therapy. The use of a continuous infusion (6 g/24 h + loading dose of 2 g) may also be used to optimise the pharmacokinetic/pharmacodynamic exposure [19]. In contrast, the dosing regimen of 2 g every 12 h may be sufficient in the treatment of uncomplicated UTI caused by carbapenem-susceptible bacteria with 3GC resistance mechanisms [9].

## Data

Data for this article are available from the Dryad Repository: https://doi.org/10.5061/dryad.931zcrjkc [20]

## Notes

### Funding

The investigation of the susceptibility of the test organisms to temocillin was supported by a grant from Eumedica S/A, Manage, Belgium, to Antiinfectives Intelligence GmbH.

### Competing interests

MK is a partner and CEO of Antiinfectives Intelligence GmbH, a research organization providing services to pharmaceutical companies. YP and GW declare that they have no competing interests.

## Supplementary Material

Supplementary Tables

## Figures and Tables

**Table 1:**
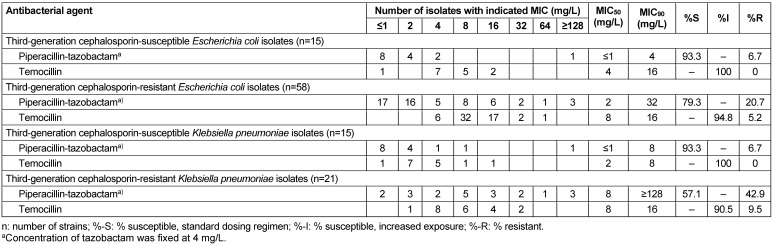
In vitro activity of piperacillin-tazobactam and temocillin

**Table 2:**
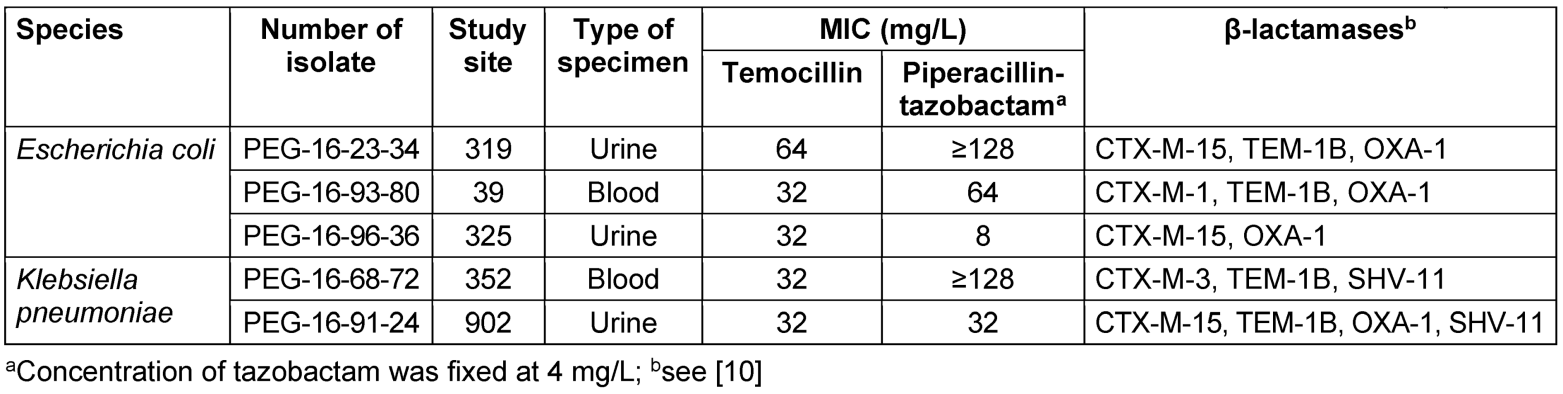
Characteristics of temocillin-resistant isolates
