# Supplementary material for: Comparative in vitro activity of piperacillin-tazobactam and temocillin against third-generation cephalosporin-resistant, carbapenem-susceptible Escherichia coli and Klebsiella pneumoniae
Source: GMS Infect Dis. 2021 Dec 21;9:Doc08. doi: 10.3205/id000077 (PMC8777484; doi:10.3205/id000077)
Supplement: Supplementary Tables [file ID-09-08-s-001.pdf]

Attachment 1: Supplementary Tables

Supplementary Table 1: In vitro activity of piperacillin-tazobactam, temocillin and comparator antibacterial agents against third-generation cephalosporin-susceptible *Escherichia coli* isolates (n=15)

**Antibacterial agent**	**MIC (mg/L)**																**MIC$_{50}$ (mg/L)**	**MIC$_{90}$ (mg/L)**	**%S**	**%I**	**%R**
	**≤0.063**	**0.125**	**0.25**	**0.5**	**1**	**2**	**4**	**8**	**16**	**32**	**64**	**128**	**256**	**512**	**1024**	**≥2048**					
Ampicillin[1]						5	2				*8*						≥64	≥64	46.7	–	53.3
Amoxicillin-clavulanic acid[2]						5	4	3	1	1		*1*					4	32	80.0	–	20.0
Amoxicillin-clavulanic acid[2] (uUTI only)						5	4	3	1	1		*1*					4	32	93.3	–	6.7
Piperacillin-tazobactam[4]					**8**	4	2					*1*					≤1	4	93.3	–	6.7
Temocillin					1		7	5	2								4	16	–	100	0
Cefuroxime[1]					1	1	10	2	1								4	8	–	93.3	6.7
Cefotaxime		**14**	1														≤0.125	≤0.125	100	0	0
Ceftriaxone		**14**	1														≤0.125	≤0.125	100	0	0
Ceftazidime			**13**	1	1												≤0.25	0.5	100	0	0
Cefepime			**14**	1													≤0.25	≤0.25	100	0	0
Imipenem				**15**													≤0.5	≤0.5	100	0	0
Meropenem	**15**																≤0.063	≤0.063	100	0	0
Ciprofloxacin	**14**								*1*								≤0.063	≤0.063	93.3	0	6.7
Levofloxacin	**14**							1									≤0.063	≤0.063	93.3	0	6.7
Amikacin					5	9		1									2	2	100	–	0
Gentamicin				11	1	2				1							0.5	2	93.3	–	6.7
Tobramycin					**14**			1									≤1	≤1	93.3	–	6.7
Colistin					**15**												≤1	≤1	100	–	0
Cotrimoxazole[4]			**11**					1		*3*							≤0.25	≥32	73.3	0	26.7
Fosfomycin[5]					**9**	2	1	1	1	1							≤1	16	100	–	0

uUTI: uncomplicated urinary tract infection; %-S: % susceptible, standard dosing regimen; %-I: % susceptible, increased exposure; %-R: % resistant.
Numbers in bold include isolates with MIC < value shown; numbers in italics include isolates with MIC > the highest concentration tested.
[1]Breakpoints are based on intravenous administration.
[2]Concentration of clavulanic acid was fixed at 2 mg/L.
[3]Concentration of tazobactam was fixed at 4 mg/L.
[4]Trimethoprim-sulfamethoxazole, MIC values are expressed as the trimethoprim concentration.
[5]Fosfomycin MICs should be viewed with caution as broth microdiltion instead of agar dilution, being the reference method, was used.

Supplementary Table 2: In vitro activity of piperacillin-tazobactam, temocillin and comparator antibacterial agents against third-generation cephalosporin-susceptible *Klebsiella pneumoniae* isolates (n=15)

**Antibacterial agent**	**MIC (mg/L)**																**MIC_{50} (mg/L)**	**MIC_{90} (mg/L)**	**%S**	**%I**	**%R**
	**≤0.063**	**0.125**	**0.25**	**0.5**	**1**	**2**	**4**	**8**	**16**	**32**	**64**	**128**	**256**	**512**	**1024**	**≥2048**					
Ampicillin[1]						1			1	10	*3*						≥64	≥64	6.7	–	93.3
Amoxicillin-clavulanic acid[2]				**5**	6	2	1					*1*					1	4	93.3	–	6.7
Amoxicillin-clavulanic acid[2] (uUTI only)						5	4	3	1	1		*1*					1	4	93.3	–	6.7
Piperacillin-tazobactam[3]					**8**	4	1	1				*1*					≤1	8	93.3	–	6.7
Temocillin					1	7	5	1	1								2	8	–	100	0
Cefuroxime[1]			1		2	6	4	1		1							2	8	–	93.3	6.7
Cefotaxime		**14**			1												≤0.125	≤0.125	100	0	0
Ceftriaxone		**14**	1														≤0.125	≤0.125	100	0	0
Ceftazidime			**13**	1	1												≤0.25	0.5	100	0	0
Cefepime			**14**	1													≤0.25	≤0.25	100	0	0
Imipenem				**14**	1												≤0.5	≤0.5	100	0	0
Meropenem	**15**																≤0.063	≤0.063	100	0	0
Ciprofloxacin	**12**		3														≤0.063	0.125	100	0	0
Levofloxacin	**9**	3	1	2													≤0.063	0.5	100	0	0
Amikacin					11	3		1									≤0.5	1	100	–	0
Gentamicin			**12**	3													≤0.25	0.5	100	––	0
Tobramycin					**14**		1										≤1	≤1	93.3	–	6.7
Colistin					**12**	1			2								≤1	≥16	86.7	–	13.3
Cotrimoxazole[4]			**10**	2	1					*2*							≤0.25	≥32	86.7	0	13.3
Fosfomycin[5]							2	1	3	4	3	2					32	128	66.7	–	33.3

uUTI: uncomplicated urinary tract infection; %-S: % susceptible, standard dosing regimen; %-I: % susceptible, increased exposure; %-R: % resistant.
Numbers in bold include isolates with MIC < value shown; numbers in italics include isolates with MIC > the highest concentration tested.
[1]Breakpoints are based on intravenous administration.
[2]Concentration of clavulanic acid was fixed at 2 mg/L.
[3]Concentration of tazobactam was fixed at 4 mg/L.
[4]Trimethoprim-sulfamethoxazole, MIC values are expressed as the trimethoprim concentration.
[5]Fosfomycin MICs should be viewed with caution as broth microdiltion instead of agar dilution, being the reference method, was used.

Supplementary Table 3: In vitro activity of piperacillin-tazobactam, temocillin and comparator antibacterial agents against third-generation cephalosporin-resistant, carbapenem-susceptible *Escherichia coli* isolates (n=58)

Antibacterial agent	MIC (mg/L)																MIC_{50} (mg/L)	MIC_{90} (mg/L)	%S	%I	%R
	**≤0.063**	**0.125**	**0.25**	**0.5**	**1**	**2**	**4**	**8**	**16**	**32**	**64**	**128**	**256**	**512**	**1024**	**≥2048**					
Ampicillin[1]											*58*						≥64	≥64	0	–	100
Amoxicillin-clavulanic acid[2]						5	12	4	12	9	8	*8*					16	≥128	36.2	–	63.8
Amoxicillin-clavulanic acid[2] (uUTI only)						5	12	4	12	9	8	*8*					16	≥128	72.4	–	27.6
Piperacillin-tazobactam[3]					**17**	16	5	8	6	2	1	*3*					2	32	79.3	–	20.7
Temocillin							6	32	17	2	1						8	16	–	94.8	5.2
Cefuroxime[1]									1	*57*							≥32	≥32	–	0	100
Cefotaxime				1			1	1	1	*54*							≥32	≥32	1.7	0	98.3
Ceftriaxone				1		1	1	0	0	*55*							≥32	≥32	1.7	1.7	96.6
Ceftazidime					3	4	8	12	13	9	*9*						16	≥64	5.2	20.7	74.1
Cefepime			**2**			2	3	5	5	6	*35*						≥64	≥64	3.4	8.6	87.9
Imipenem				**58**													≤0.5	≤0.5	100	0	0
Meropenem	**58**																≤0.063	≤0.063	100	0	0
Ciprofloxacin	**9**	1	2	3	1			2	*40*								≥16	≥16	20.7	5.2	74.1
Levofloxacin	**8**	1	1	5		1	3	23	*16*								8	≥16	25.9	0	74.1
Amikacin				**1**	15	21	16	3	2								2	4	96.6	–	3.4
Gentamicin				27	11	2		3	1	*14*							1	≥32	69.0	–	31.0
Tobramycin					**32**	3	3	8	*12*								≤1	≥16	60.3	–	39.7
Colistin					**55**	2	1										≤1	≤1	98.3	–	1.7
Cotrimoxazole[4]			**15**	1	1					*41*							≥32	≥32	29.3	0	70.7
Fosfomycin[5]					**15**	17	13	8	3		1			1			2	8	96.6	–	3.4

Resistance to third-generation cephalosporin was defined as resistance to cefotaxime (MIC>2 mg/L) and/or resistance to ceftazidime (MIC>4 mg/L).
uUTI: uncomplicated urinary tract infection; %-S: % susceptible, standard dosing regimen; %-I: % susceptible, increased exposure; %-R: % resistant.
Numbers in bold include isolates with MIC < value shown; numbers in italics include isolates with MIC > the highest concentration tested.
[1]Breakpoints are based on intravenous administration.
[2]Concentration of clavulanic acid was fixed at 2 mg/L.
[3]Concentration of tazobactam was fixed at 4 mg/L.
[4]Trimethoprim-sulfamethoxazole, MIC values are expressed as the trimethoprim concentration.
[5]Fosfomycin MICs should be viewed with caution as broth microdiltion instead of agar dilution, being the reference method, was used.

Supplementary Table 4: In vitro activity of piperacillin-tazobactam, temocillin and comparator antibacterial agents against third-generation cephalosporin-resistant, carbapenem-susceptible *Klebsiella pneumoniae* isolates (n=21)

Antibacterial agent	MIC (mg/L)																MIC50 (mg/L)	MIC90 (mg/L)	%S	%I	%R
	≤0.063	0.125	0.25	0.5	1	2	4	8	16	32	64	128	256	512	1024	≥2048					
Ampicillin[1]											*21*						≥64	≥64	0	–	100
Amoxicillin-clavulanic acid[2]						1	1	3	2	4	6	*4*					32	≥128	23.8	–	76.2
Amoxicillin-clavulanic acid[2] (uUTI only)						1	1	3	2	4	6	*4*					32	≥128	52.4	–	47.6
Piperacillin-tazobactam[3]					**2**	3	2	5	3	2	1	*3*					8	≥128	57.1	–	42.9
Temocillin						1	8	6	4	2							8	16	–	90.5	9.5
Cefuroxime[1]									1	*20*							≥32	≥32	0	–	100
Cefotaxime			1						1	*19*							≥32	≥32	4.8	0	95.2
Ceftriaxone				1					1	*19*							≥32	≥32	4.8	0	96.6
Ceftazidime							1	3	5	5	*7*						32	≥64	0	4.8	95.2
Cefepime						2		3	1	3	*12*						≥64	≥64	0	9.5	90.5
Imipenem				**21**													≤0.5	≤0.5	100	0	0
Meropenem	**21**																≤0.063	≤0.063	100	0	0
Ciprofloxacin				6	1	3	2		*9*								4	≥16	0	28.6	71.4
Levofloxacin				7	3		8	2	*1*								4	8	33.3	14.3	52.4
Amikacin (UTI only)				**6**	12	2	1										1	2	100	–	0
Gentamicin			13	2					3	*3*							≤0.25	≥32	71.4	–	28.6
Tobramycin					**9**	1	8	3									4	8	47.6	–	52.4
Colistin					**20**				*1*								≤1	≤1	95.2	–	4.8
Cotrimoxazole[4]			**1**		1					*19*							≥32	≥32	9.5	0	90.5
Fosfomycin[5]								3	2	4	6	3	1	1		*1*	64	256	42.9	–	57.1

Resistance to third-generation cephalosporin was defined as resistance to cefotaxime (MIC>2 mg/L) and/or resistance to ceftazidime (MIC>4 mg/L).
uUTI: uncomplicated urinary tract infection; %-S: % susceptible, standard dosing regimen; %-I: % susceptible, increased exposure; %-R: % resistant.
Numbers in bold include isolates with MIC < value shown; numbers in italics include isolates with MIC > the highest concentration tested.
[1]Breakpoints are based on intravenous administration.
[2]Concentration of clavulanic acid was fixed at 2 mg/L.
[3]Concentration of tazobactam was fixed at 4 mg/L.
[4]Trimethoprim-sulfamethoxazole, MIC values are expressed as the trimethoprim concentration.
[5]Fosfomycin MICs should be viewed with caution as broth microdiltion instead of agar dilution, being the reference method, was used.
